# 2-Eth­oxy-6-[1-(3-eth­oxy-2-hy­droxy­benz­yl)-2,3-dihydro-1*H*-benzimidazol-2-yl]phenol acetonitrile monosolvate

**DOI:** 10.1107/S1600536812015449

**Published:** 2012-04-18

**Authors:** Kwang Ha

**Affiliations:** a School of Applied Chemical Engineering, The Research Institute of Catalysis, Chonnam National University, Gwangju 500-757, Republic of Korea

## Abstract

The title compound, C_24_H_24_N_2_O_4_·CH_3_CN, a disubstituted benzimidazole, crystallized as an acetonitrile monosolvate. The benzene ring of the 2-eth­oxy-6-methyl­phenol substiuent is approximately perpendicular to the nearly planar benzimidazole ring system [maximum deviation = 0.016 (1) Å], making a dihedral angle of 84.27 (8)°. The benzene ring of the 2-eth­oxy­phenol substituent is inclined to the benzimidazole mean plane by 29.68 (8)°. The dihedral angle between the benzene rings is 80.36 (9)°. In the mol­ecule, there are strong O—H⋯N and O—H⋯O hydrogen bonds. In the crystal, mol­ecules are connected by bifurcated O—H⋯(O,O) hydrogen bonds, forming chains propagating along [010].

## Related literature

For the crystal structure of the meth­oxy derivative of the title compound, see: Al-Douh *et al.* (2009 ▶).
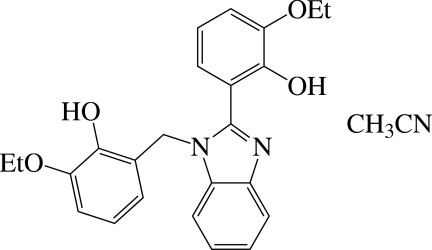


## Experimental

### 

#### Crystal data

C_24_H_24_N_2_O_4_·C_2_H_3_N
*M* *_r_* = 445.51
Monoclinic, 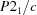

*a* = 7.6177 (3) Å
*b* = 19.2728 (8) Å
*c* = 16.3480 (7) Å
β = 99.991 (1)°
*V* = 2363.72 (17) Å^3^
*Z* = 4
Mo *K*α radiation
μ = 0.09 mm^−1^
*T* = 200 K
0.27 × 0.24 × 0.20 mm

#### Data collection

Bruker SMART 1000 CCD diffractometer
Absorption correction: multi-scan (*SADABS*; Bruker, 2000 ▶) *T*_min_ = 0.853, *T*_max_ = 1.000
17475 measured reflections
5852 independent reflections
2912 reflections with *I* > 2σ(*I*)
*R*_int_ = 0.059

#### Refinement

*R*[*F* ^2^ > 2σ(*F* ^2^)] = 0.049
*wR*(*F* ^2^) = 0.129
*S* = 0.96
5852 reflections
309 parameters
H atoms treated by a mixture of independent and constrained refinement
Δρ_max_ = 0.20 e Å^−3^
Δρ_min_ = −0.24 e Å^−3^

### 

Data collection: *SMART* (Bruker, 2000 ▶); cell refinement: *SAINT* (Bruker, 2000 ▶); data reduction: *SAINT*; program(s) used to solve structure: *SHELXS97* (Sheldrick, 2008 ▶); program(s) used to refine structure: *SHELXL97* (Sheldrick, 2008 ▶); molecular graphics: *ORTEP-3* (Farrugia, 1997 ▶) and *PLATON* (Spek, 2009 ▶); software used to prepare material for publication: *SHELXL97*.

## Supplementary Material

Crystal structure: contains datablock(s) global, I. DOI: 10.1107/S1600536812015449/su2405sup1.cif

Structure factors: contains datablock(s) I. DOI: 10.1107/S1600536812015449/su2405Isup2.hkl

Supplementary material file. DOI: 10.1107/S1600536812015449/su2405Isup3.cml

Additional supplementary materials:  crystallographic information; 3D view; checkCIF report

## Figures and Tables

**Table 1:** Hydrogen-bond geometry (Å, °)

*D*—H⋯*A*	*D*—H	H⋯*A*	*D*⋯*A*	*D*—H⋯*A*
O1—H1*O*⋯N1	0.95 (3)	1.72 (3)	2.602 (2)	152 (2)
O3—H3*O*⋯O4	0.94 (3)	2.33 (3)	2.7180 (18)	103.9 (18)
O3—H3*O*⋯O1^i^	0.94 (3)	1.89 (3)	2.7774 (19)	158 (2)
O3—H3*O*⋯O2^i^	0.94 (3)	2.43 (3)	3.0117 (19)	119.7 (19)

Symmetry code: (i) 
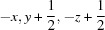
.

**Fig. 1.**
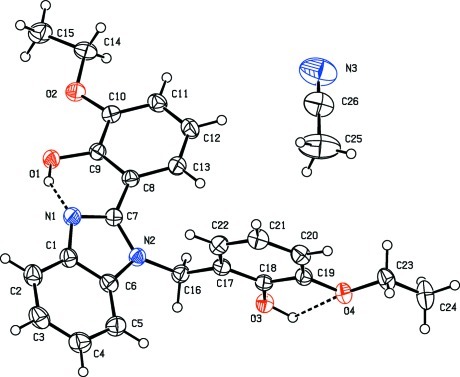
The molecular structure of the title compound, with atom numbering. Displacement ellipsoids are drawn at the 40% probability level. Intramolecular hydrogen-bonds are shown as dashed lines (see Table 1 for details).

**Fig. 2.**
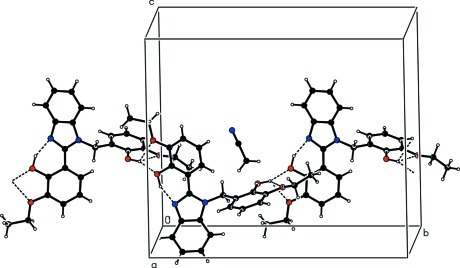
A partial view along the a axis of the crystal packing of the title compound. Intra- and intermolecular O—H···N and O—H···O hydrogen-bonds are shown as dashed lines (see Table 1 for details).

## supplementary crystallographic information

### Comment

The crystal structure of the related compound, 2-methoxy-6-(1-(3-methoxy-2-hydroxybenzyl)-2,3-dihydro-1H-benzo[d]imidazol-2-yl)phenol monohydrate, has been reported on previously (Al-Douh *et al.*, 2009).

The title compound contains a disubstituted benzimidazole molecule and a lattice solvent molecule (Fig. 1). One of the benzene rings (C17-C22) is approximately perpendicular to the nearly planar benzimidazole ring system [maximum deviation = 0.016 (1) Å], making a dihedral angle of 84.27 (8)°, whereas the other ring (C8-C13) is somewhat inclined to the benzomidazole mean plane with a dihedral angle of 29.68 (8)°. The dihedral angle between the benzene rings is 80.36 (9)°. The compound reveals strong intramolecular O—H···N and O—H···O hydrogen bonds, forming six- and five-membered rings, respectively (Fig. 1 and Table 1).

In the crystal, molecules are connected by bifurcated O—H···O,O hydrogen bonds, forming chains along the *b* axis (Fig. 2 and Table 1).

### Experimental

1,2-Phenylenediamine (0.7568 g, 6.998 mmol) and 3-ethoxysalicylaldehyde (2.3269 g, 14.003 mmol) in EtOH (20 ml) were stirred for 5 h at room temperature. After evaporation of the solvent, the residue was recrystallized from a mixture of acetone and ether (1:2, v:v) at 188 K, to give an orange powder (1.9139 g). Orange block-like crystals, suitable for X-ray analysis, were obtained by slow evaporation of a CH_3_CN/acetone solution at room temperature.

### Refinement

The hydroxy H atoms were located from a difference Fourier map and refined freely. C-bound H atoms were included in calculated positions and treated as riding atoms: C—H = 0.95, 0.98 and 0.99 Å for CH, CH_3_ and CH_2_ H-atoms, respectively, with U_iso_(H) = k× U_eq_(parent C-atom), where k = 1.5 for CH_3_ H-atoms and = 1.2 for other H-atoms.

### Crystal data

**Table:**

C_24_H_24_N_2_O_4_·C_2_H_3_N	*F*(000) = 944
*M**_r_* = 445.51	*D*_x_ = 1.252 Mg m^−^^3^
Monoclinic, *P*2_1_/*c*	Mo *K*α radiation, λ = 0.71073 Å
Hall symbol: -P 2ybc	Cell parameters from 3400 reflections
*a* = 7.6177 (3) Å	θ = 2.5–26.4°
*b* = 19.2728 (8) Å	µ = 0.09 mm^−^^1^
*c* = 16.3480 (7) Å	*T* = 200 K
β = 99.991 (1)°	Block, orange
*V* = 2363.72 (17) Å^3^	0.27 × 0.24 × 0.20 mm
*Z* = 4	

### Data collection

**Table:**

Bruker SMART 1000 CCD diffractometer	5852 independent reflections
Radiation source: fine-focus sealed tube	2912 reflections with *I* > 2σ(*I*)
Graphite monochromator	*R*_int_ = 0.059
φ and ω scans	θ_max_ = 28.3°, θ_min_ = 2.1°
Absorption correction: multi-scan (*SADABS*; Bruker, 2000)	*h* = −8→10
*T*_min_ = 0.853, *T*_max_ = 1.000	*k* = −25→24
17475 measured reflections	*l* = −21→21

### Refinement

**Table:**

Refinement on *F*^2^	Primary atom site location: structure-invariant direct methods
Least-squares matrix: full	Secondary atom site location: difference Fourier map
*R*[*F*^2^ > 2σ(*F*^2^)] = 0.049	Hydrogen site location: inferred from neighbouring sites
*wR*(*F*^2^) = 0.129	H atoms treated by a mixture of independent and constrained refinement
*S* = 0.96	*w* = 1/[σ^2^(*F*_o_^2^) + (0.0484*P*)^2^] where *P* = (*F*_o_^2^ + 2*F*_c_^2^)/3
5852 reflections	(Δ/σ)_max_ < 0.001
309 parameters	Δρ_max_ = 0.20 e Å^−^^3^
0 restraints	Δρ_min_ = −0.24 e Å^−^^3^

### Special details

**Table:**

Geometry. All e.s.d.'s (except the e.s.d. in the dihedral angle between two l.s. planes) are estimated using the full covariance matrix. The cell e.s.d.'s are taken into account individually in the estimation of e.s.d.'s in distances, angles and torsion angles; correlations between e.s.d.'s in cell parameters are only used when they are defined by crystal symmetry. An approximate (isotropic) treatment of cell e.s.d.'s is used for estimating e.s.d.'s involving l.s. planes.
Refinement. Refinement of *F*^2^ against ALL reflections. The weighted *R*-factor *wR* and goodness of fit *S* are based on *F*^2^, conventional *R*-factors *R* are based on *F*, with *F* set to zero for negative *F*^2^. The threshold expression of *F*^2^ > σ(*F*^2^) is used only for calculating *R*-factors(gt) *etc*. and is not relevant to the choice of reflections for refinement. *R*-factors based on *F*^2^ are statistically about twice as large as those based on *F*, and *R*- factors based on ALL data will be even larger.

### Fractional atomic coordinates and isotropic or equivalent isotropic displacement parameters (Å^2^)

**Table:**

	*x*	*y*	*z*	*U*_iso_*/*U*_eq_	
O1	0.15735 (18)	−0.00918 (6)	0.26012 (8)	0.0365 (3)	
H1O	0.168 (3)	0.0028 (13)	0.2049 (17)	0.094 (9)*	
O2	0.19475 (18)	−0.02073 (7)	0.41963 (7)	0.0417 (4)	
O3	−0.00142 (17)	0.37008 (7)	0.19346 (8)	0.0365 (3)	
H3O	−0.037 (3)	0.4162 (14)	0.1998 (15)	0.096 (9)*	
O4	0.22786 (17)	0.47927 (7)	0.21034 (8)	0.0394 (3)	
N1	0.2178 (2)	0.06036 (8)	0.13143 (9)	0.0379 (4)	
N2	0.1411 (2)	0.17258 (8)	0.12832 (9)	0.0342 (4)	
C1	0.1805 (3)	0.08206 (10)	0.04922 (11)	0.0362 (5)	
C2	0.1843 (3)	0.04599 (11)	−0.02431 (12)	0.0443 (5)	
H2	0.2157	−0.0017	−0.0237	0.053*	
C3	0.1412 (3)	0.08162 (12)	−0.09760 (12)	0.0491 (6)	
H3	0.1431	0.0580	−0.1485	0.059*	
C4	0.0946 (3)	0.15150 (12)	−0.09935 (13)	0.0505 (6)	
H4	0.0658	0.1744	−0.1514	0.061*	
C5	0.0892 (3)	0.18836 (11)	−0.02765 (12)	0.0442 (5)	
H5	0.0574	0.2361	−0.0287	0.053*	
C6	0.1330 (2)	0.15182 (10)	0.04647 (11)	0.0358 (5)	
C7	0.1956 (2)	0.11603 (10)	0.17655 (11)	0.0329 (4)	
C8	0.2259 (2)	0.11358 (10)	0.26759 (11)	0.0320 (4)	
C9	0.2022 (2)	0.04994 (9)	0.30469 (11)	0.0316 (4)	
C10	0.2278 (2)	0.04394 (10)	0.39178 (11)	0.0343 (5)	
C11	0.2845 (3)	0.10069 (10)	0.44051 (12)	0.0395 (5)	
H11	0.3030	0.0969	0.4993	0.047*	
C12	0.3147 (3)	0.16344 (11)	0.40360 (12)	0.0433 (5)	
H12	0.3562	0.2021	0.4375	0.052*	
C13	0.2854 (3)	0.17026 (10)	0.31876 (12)	0.0392 (5)	
H13	0.3055	0.2137	0.2945	0.047*	
C14	0.2317 (3)	−0.03357 (11)	0.50729 (11)	0.0452 (5)	
H14A	0.1566	−0.0036	0.5363	0.054*	
H14B	0.3585	−0.0238	0.5298	0.054*	
C15	0.1904 (3)	−0.10855 (11)	0.51931 (13)	0.0543 (6)	
H15A	0.0655	−0.1177	0.4955	0.081*	
H15B	0.2110	−0.1193	0.5788	0.081*	
H15C	0.2678	−0.1376	0.4915	0.081*	
C16	0.0792 (2)	0.23988 (9)	0.15229 (11)	0.0355 (5)	
H16A	0.0403	0.2352	0.2068	0.043*	
H16B	−0.0259	0.2540	0.1111	0.043*	
C17	0.2181 (2)	0.29633 (10)	0.15836 (11)	0.0323 (4)	
C18	0.1690 (2)	0.36206 (9)	0.17971 (10)	0.0304 (4)	
C19	0.2916 (3)	0.41653 (10)	0.18826 (11)	0.0335 (4)	
C20	0.4630 (3)	0.40455 (11)	0.17447 (11)	0.0405 (5)	
H20	0.5469	0.4414	0.1797	0.049*	
C21	0.5126 (3)	0.33855 (11)	0.15293 (12)	0.0427 (5)	
H21	0.6306	0.3304	0.1438	0.051*	
C22	0.3911 (3)	0.28487 (11)	0.14468 (11)	0.0393 (5)	
H22	0.4256	0.2399	0.1296	0.047*	
C23	0.3560 (3)	0.53233 (11)	0.23731 (13)	0.0468 (6)	
H23A	0.4117	0.5482	0.1902	0.056*	
H23B	0.4507	0.5141	0.2812	0.056*	
C24	0.2611 (3)	0.59180 (12)	0.27053 (15)	0.0630 (7)	
H24A	0.1700	0.6104	0.2262	0.094*	
H24B	0.3472	0.6283	0.2907	0.094*	
H24C	0.2043	0.5754	0.3163	0.094*	
N3	0.6545 (4)	0.31138 (16)	0.53251 (17)	0.1080 (10)	
C25	0.6024 (4)	0.3616 (2)	0.38529 (19)	0.1163 (13)	
H25A	0.6354	0.4108	0.3874	0.174*	
H25B	0.4764	0.3568	0.3605	0.174*	
H25C	0.6758	0.3363	0.3516	0.174*	
C26	0.6315 (4)	0.33353 (16)	0.46845 (19)	0.0758 (8)	

### Atomic displacement parameters (Å^2^)

**Table:**

	*U*^11^	*U*^22^	*U*^33^	*U*^12^	*U*^13^	*U*^23^
O1	0.0473 (9)	0.0318 (8)	0.0306 (8)	−0.0014 (6)	0.0072 (6)	−0.0023 (6)
O2	0.0560 (9)	0.0414 (9)	0.0282 (7)	−0.0007 (7)	0.0087 (6)	0.0049 (6)
O3	0.0346 (8)	0.0348 (9)	0.0412 (8)	−0.0011 (6)	0.0095 (6)	−0.0018 (6)
O4	0.0410 (8)	0.0360 (8)	0.0417 (8)	−0.0088 (6)	0.0084 (6)	−0.0066 (6)
N1	0.0495 (11)	0.0350 (10)	0.0291 (9)	0.0019 (8)	0.0066 (8)	−0.0022 (7)
N2	0.0423 (10)	0.0307 (9)	0.0302 (9)	0.0003 (7)	0.0082 (7)	−0.0012 (7)
C1	0.0404 (12)	0.0372 (12)	0.0321 (11)	−0.0035 (9)	0.0097 (9)	−0.0029 (9)
C2	0.0547 (14)	0.0426 (13)	0.0372 (12)	−0.0034 (10)	0.0122 (10)	−0.0070 (10)
C3	0.0577 (15)	0.0587 (16)	0.0322 (12)	−0.0089 (12)	0.0115 (10)	−0.0078 (11)
C4	0.0626 (16)	0.0561 (15)	0.0334 (12)	−0.0045 (12)	0.0101 (11)	0.0046 (11)
C5	0.0540 (14)	0.0424 (13)	0.0372 (12)	−0.0014 (10)	0.0101 (10)	0.0050 (10)
C6	0.0368 (12)	0.0408 (12)	0.0303 (11)	−0.0036 (9)	0.0075 (9)	−0.0012 (9)
C7	0.0342 (11)	0.0329 (11)	0.0321 (10)	−0.0008 (8)	0.0074 (8)	−0.0001 (9)
C8	0.0331 (11)	0.0339 (11)	0.0291 (10)	0.0027 (8)	0.0054 (8)	−0.0017 (9)
C9	0.0306 (11)	0.0320 (11)	0.0321 (11)	0.0031 (8)	0.0054 (8)	−0.0036 (9)
C10	0.0337 (11)	0.0364 (12)	0.0336 (11)	0.0021 (9)	0.0077 (9)	0.0025 (9)
C11	0.0445 (13)	0.0454 (13)	0.0283 (10)	0.0009 (10)	0.0055 (9)	−0.0035 (10)
C12	0.0504 (14)	0.0435 (13)	0.0346 (12)	−0.0035 (10)	0.0038 (10)	−0.0085 (10)
C13	0.0462 (13)	0.0345 (12)	0.0360 (12)	−0.0001 (9)	0.0049 (9)	0.0002 (9)
C14	0.0480 (13)	0.0582 (15)	0.0291 (11)	0.0000 (11)	0.0059 (9)	0.0066 (10)
C15	0.0678 (16)	0.0567 (16)	0.0393 (13)	0.0015 (12)	0.0117 (11)	0.0119 (11)
C16	0.0402 (12)	0.0327 (11)	0.0341 (11)	0.0021 (9)	0.0076 (9)	0.0001 (9)
C17	0.0362 (12)	0.0348 (11)	0.0266 (10)	−0.0001 (9)	0.0070 (8)	0.0011 (8)
C18	0.0323 (11)	0.0343 (11)	0.0247 (10)	0.0004 (9)	0.0057 (8)	0.0019 (8)
C19	0.0401 (12)	0.0331 (11)	0.0276 (10)	−0.0020 (9)	0.0069 (9)	−0.0009 (8)
C20	0.0399 (13)	0.0455 (13)	0.0367 (11)	−0.0101 (10)	0.0080 (9)	0.0018 (10)
C21	0.0385 (12)	0.0499 (14)	0.0416 (12)	0.0014 (10)	0.0122 (10)	−0.0003 (10)
C22	0.0450 (13)	0.0377 (12)	0.0366 (11)	0.0030 (10)	0.0107 (9)	0.0010 (9)
C23	0.0457 (13)	0.0437 (13)	0.0492 (13)	−0.0145 (10)	0.0033 (10)	−0.0089 (11)
C24	0.0644 (16)	0.0475 (15)	0.0793 (18)	−0.0154 (12)	0.0191 (14)	−0.0244 (13)
N3	0.091 (2)	0.155 (3)	0.0782 (19)	−0.0154 (17)	0.0162 (16)	0.0298 (18)
C25	0.083 (2)	0.184 (4)	0.081 (2)	0.012 (2)	0.0128 (18)	0.048 (2)
C26	0.0613 (18)	0.099 (2)	0.067 (2)	−0.0066 (15)	0.0124 (15)	0.0110 (17)

### Geometric parameters (Å, º)

**Table:**

O1—C9	1.364 (2)	C12—H12	0.9500
O1—H1O	0.95 (3)	C13—H13	0.9500
O2—C10	1.365 (2)	C14—C15	1.499 (3)
O2—C14	1.433 (2)	C14—H14A	0.9900
O3—C18	1.364 (2)	C14—H14B	0.9900
O3—H3O	0.94 (3)	C15—H15A	0.9800
O4—C19	1.375 (2)	C15—H15B	0.9800
O4—C23	1.429 (2)	C15—H15C	0.9800
N1—C7	1.329 (2)	C16—C17	1.509 (2)
N1—C1	1.389 (2)	C16—H16A	0.9900
N2—C7	1.367 (2)	C16—H16B	0.9900
N2—C6	1.388 (2)	C17—C18	1.383 (2)
N2—C16	1.457 (2)	C17—C22	1.392 (2)
C1—C6	1.391 (3)	C18—C19	1.396 (2)
C1—C2	1.393 (3)	C19—C20	1.382 (3)
C2—C3	1.370 (3)	C20—C21	1.390 (3)
C2—H2	0.9500	C20—H20	0.9500
C3—C4	1.392 (3)	C21—C22	1.379 (3)
C3—H3	0.9500	C21—H21	0.9500
C4—C5	1.377 (3)	C22—H22	0.9500
C4—H4	0.9500	C23—C24	1.506 (3)
C5—C6	1.391 (3)	C23—H23A	0.9900
C5—H5	0.9500	C23—H23B	0.9900
C7—C8	1.467 (2)	C24—H24A	0.9800
C8—C9	1.394 (2)	C24—H24B	0.9800
C8—C13	1.402 (3)	C24—H24C	0.9800
C9—C10	1.408 (2)	N3—C26	1.116 (3)
C10—C11	1.378 (3)	C25—C26	1.444 (4)
C11—C12	1.388 (3)	C25—H25A	0.9800
C11—H11	0.9500	C25—H25B	0.9800
C12—C13	1.372 (3)	C25—H25C	0.9800
			
C9—O1—H1O	104.5 (15)	C15—C14—H14B	110.4
C10—O2—C14	118.39 (15)	H14A—C14—H14B	108.6
C18—O3—H3O	115.1 (16)	C14—C15—H15A	109.5
C19—O4—C23	117.18 (15)	C14—C15—H15B	109.5
C7—N1—C1	105.62 (15)	H15A—C15—H15B	109.5
C7—N2—C6	106.53 (15)	C14—C15—H15C	109.5
C7—N2—C16	129.40 (15)	H15A—C15—H15C	109.5
C6—N2—C16	123.63 (15)	H15B—C15—H15C	109.5
N1—C1—C6	109.32 (16)	N2—C16—C17	113.79 (15)
N1—C1—C2	130.78 (19)	N2—C16—H16A	108.8
C6—C1—C2	119.90 (18)	C17—C16—H16A	108.8
C3—C2—C1	117.8 (2)	N2—C16—H16B	108.8
C3—C2—H2	121.1	C17—C16—H16B	108.8
C1—C2—H2	121.1	H16A—C16—H16B	107.7
C2—C3—C4	121.63 (19)	C18—C17—C22	119.42 (17)
C2—C3—H3	119.2	C18—C17—C16	117.35 (16)
C4—C3—H3	119.2	C22—C17—C16	123.22 (17)
C5—C4—C3	121.8 (2)	O3—C18—C17	116.90 (16)
C5—C4—H4	119.1	O3—C18—C19	122.53 (17)
C3—C4—H4	119.1	C17—C18—C19	120.55 (17)
C4—C5—C6	116.2 (2)	O4—C19—C20	125.22 (17)
C4—C5—H5	121.9	O4—C19—C18	115.26 (16)
C6—C5—H5	121.9	C20—C19—C18	119.52 (18)
N2—C6—C1	106.24 (16)	C19—C20—C21	120.01 (18)
N2—C6—C5	131.13 (19)	C19—C20—H20	120.0
C1—C6—C5	122.63 (18)	C21—C20—H20	120.0
N1—C7—N2	112.25 (16)	C22—C21—C20	120.29 (19)
N1—C7—C8	121.61 (17)	C22—C21—H21	119.9
N2—C7—C8	126.14 (16)	C20—C21—H21	119.9
C9—C8—C13	118.59 (17)	C21—C22—C17	120.20 (19)
C9—C8—C7	117.41 (16)	C21—C22—H22	119.9
C13—C8—C7	123.92 (17)	C17—C22—H22	119.9
O1—C9—C8	122.82 (16)	O4—C23—C24	108.13 (17)
O1—C9—C10	116.72 (16)	O4—C23—H23A	110.1
C8—C9—C10	120.44 (17)	C24—C23—H23A	110.1
O2—C10—C11	126.11 (17)	O4—C23—H23B	110.1
O2—C10—C9	114.27 (16)	C24—C23—H23B	110.1
C11—C10—C9	119.61 (18)	H23A—C23—H23B	108.4
C10—C11—C12	119.94 (18)	C23—C24—H24A	109.5
C10—C11—H11	120.0	C23—C24—H24B	109.5
C12—C11—H11	120.0	H24A—C24—H24B	109.5
C13—C12—C11	120.82 (19)	C23—C24—H24C	109.5
C13—C12—H12	119.6	H24A—C24—H24C	109.5
C11—C12—H12	119.6	H24B—C24—H24C	109.5
C12—C13—C8	120.50 (19)	C26—C25—H25A	109.5
C12—C13—H13	119.8	C26—C25—H25B	109.5
C8—C13—H13	119.8	H25A—C25—H25B	109.5
O2—C14—C15	106.75 (16)	C26—C25—H25C	109.5
O2—C14—H14A	110.4	H25A—C25—H25C	109.5
C15—C14—H14A	110.4	H25B—C25—H25C	109.5
O2—C14—H14B	110.4	N3—C26—C25	179.5 (4)
			
C7—N1—C1—C6	−0.8 (2)	O1—C9—C10—O2	−3.2 (2)
C7—N1—C1—C2	179.1 (2)	C8—C9—C10—O2	178.09 (16)
N1—C1—C2—C3	−179.59 (19)	O1—C9—C10—C11	175.79 (16)
C6—C1—C2—C3	0.4 (3)	C8—C9—C10—C11	−2.9 (3)
C1—C2—C3—C4	−0.1 (3)	O2—C10—C11—C12	179.33 (18)
C2—C3—C4—C5	−0.1 (3)	C9—C10—C11—C12	0.5 (3)
C3—C4—C5—C6	0.1 (3)	C10—C11—C12—C13	1.3 (3)
C7—N2—C6—C1	1.2 (2)	C11—C12—C13—C8	−0.7 (3)
C16—N2—C6—C1	−171.89 (16)	C9—C8—C13—C12	−1.7 (3)
C7—N2—C6—C5	−178.6 (2)	C7—C8—C13—C12	−178.45 (18)
C16—N2—C6—C5	8.3 (3)	C10—O2—C14—C15	−177.74 (16)
N1—C1—C6—N2	−0.3 (2)	C7—N2—C16—C17	101.5 (2)
C2—C1—C6—N2	179.79 (17)	C6—N2—C16—C17	−87.1 (2)
N1—C1—C6—C5	179.54 (17)	N2—C16—C17—C18	179.13 (15)
C2—C1—C6—C5	−0.4 (3)	N2—C16—C17—C22	−1.7 (2)
C4—C5—C6—N2	179.94 (19)	C22—C17—C18—O3	−179.15 (15)
C4—C5—C6—C1	0.2 (3)	C16—C17—C18—O3	0.0 (2)
C1—N1—C7—N2	1.6 (2)	C22—C17—C18—C19	−0.5 (3)
C1—N1—C7—C8	−179.11 (16)	C16—C17—C18—C19	178.61 (16)
C6—N2—C7—N1	−1.8 (2)	C23—O4—C19—C20	−13.3 (3)
C16—N2—C7—N1	170.74 (17)	C23—O4—C19—C18	166.75 (16)
C6—N2—C7—C8	178.97 (17)	O3—C18—C19—O4	−0.9 (2)
C16—N2—C7—C8	−8.5 (3)	C17—C18—C19—O4	−179.46 (15)
N1—C7—C8—C9	−27.4 (3)	O3—C18—C19—C20	179.11 (16)
N2—C7—C8—C9	151.77 (18)	C17—C18—C19—C20	0.6 (3)
N1—C7—C8—C13	149.37 (19)	O4—C19—C20—C21	179.54 (17)
N2—C7—C8—C13	−31.5 (3)	C18—C19—C20—C21	−0.5 (3)
C13—C8—C9—O1	−175.13 (17)	C19—C20—C21—C22	0.4 (3)
C7—C8—C9—O1	1.8 (3)	C20—C21—C22—C17	−0.4 (3)
C13—C8—C9—C10	3.5 (3)	C18—C17—C22—C21	0.4 (3)
C7—C8—C9—C10	−179.53 (16)	C16—C17—C22—C21	−178.67 (17)
C14—O2—C10—C11	−4.1 (3)	C19—O4—C23—C24	−171.40 (16)
C14—O2—C10—C9	174.76 (16)		

### Hydrogen-bond geometry (Å, º)

**Table:**

*D*—H···*A*	*D*—H	H···*A*	*D*···*A*	*D*—H···*A*
O1—H1*O*···N1	0.95 (3)	1.72 (3)	2.602 (2)	152 (2)
O3—H3*O*···O4	0.94 (3)	2.33 (3)	2.7180 (18)	103.9 (18)
O3—H3*O*···O1^i^	0.94 (3)	1.89 (3)	2.7774 (19)	158 (2)
O3—H3*O*···O2^i^	0.94 (3)	2.43 (3)	3.0117 (19)	119.7 (19)

Symmetry code: (i) −*x*, *y*+1/2, −*z*+1/2.
